# A Complex Multiherbal Regimen Based on Ayurveda Medicine for the Management of Hepatic Cirrhosis Complicated by Ascites: Nonrandomized, Uncontrolled, Single Group, Open-Label Observational Clinical Study

**DOI:** 10.1155/2015/613182

**Published:** 2015-08-03

**Authors:** Manish V. Patel, Kalapi B. Patel, Shivenarain Gupta, Andreas Michalsen, Elmar Stapelfeldt, Christian S. Kessler

**Affiliations:** ^1^Department of Kayachikitsa, J. S. Ayurved College, College Road, Nadiad 387001, India; ^2^Department of Panchakarma, J. S. Ayurved College, College Road, Nadiad 387001, India; ^3^Department of Internal and Complementary Medicine, Immanuel Hospital and Institute of Social Medicine, Epidemiology & Health Economics, Charité-University Medical Center, Königstrasse 63, 14109 Berlin, Germany

## Abstract

Hepatic cirrhosis is one of the leading causes of death worldwide, especially if complicated by ascites. This chronic condition can be related to the classical disease entity *jalodara* in Traditional Indian Medicine (Ayurveda). The present paper aims to evaluate the general potential of Ayurvedic therapy for overall clinical outcomes in hepatic cirrhosis complicated by ascites (HCcA). In form of a nonrandomized, uncontrolled, single group, open-label observational clinical study, 56 patients fulfilling standardized diagnostic criteria for HCcA were observed during their treatment at the P. D. Patel Ayurveda Hospital, Nadiad, India. Based on Ayurvedic tradition, a standardized treatment protocol was developed and implemented, consisting of oral administration of single and compound herbal preparations combined with purificatory measures as well as dietary and lifestyle regimens. The outcomes were assessed by measuring liver functions through specific clinical features and laboratory parameters and by evaluating the Child-Pugh prognostic grade score. After 6 weeks of treatment and a follow-up period of 18 weeks, the outcomes showed statistically significant and clinically relevant improvements. Further larger and randomized trials on effectiveness, safety, and quality of the Ayurvedic approach in the treatment of HCcA are warranted to support these preliminary findings.

## 1. Introduction

Hepatic cirrhosis, especially if complicated by ascites, causes remarkable damage in human health and lives. Its management involves high costs for health care systems worldwide. Liver transplantation as one of the few treatment options bears risks and is largely unavailable or unaffordable for common patients in many countries, particularly in India. Therefore, alternative treatment measures are desirable.

Due to the high prevalence of chronic hepatic diseases in South Asia, Traditional Indian Medicine (Ayurveda) has generated extensive empirical knowledge in their treatment over several centuries.

In addition to observations of successfully treated cases in Ayurvedic clinics, preliminary scientific findings suggest that an exploration of traditional Ayurvedic literature and commonly implemented Ayurvedic treatment modalities might be worthwhile in this field.

*Hepatic Cirrhosis—Epidemiological Data Worldwide*. Hepatic cirrhosis represents the end stage of most chronic liver diseases, which can remain compensated for many years [1]. Decompensated cirrhosis can be characterized by the development of major complications like jaundice, variceal hemorrhage, ascites, or encephalopathy [2], of which ascites is the most common [3]. Approximately 50% of patients with compensated cirrhosis develop ascites over a period of 10 years [4]. The occurrence of ascites is often a landmark in the natural progress of cirrhosis, as it requires hospitalization and many patients are referred for liver transplantation [3, 5–7]. In this sense, the disease carries the risk of life-threatening complications, partly due to a number of comorbidities. Established cirrhosis has a 10-year mortality of 34–66%, being largely dependent on the cause of cirrhosis; alcoholic cirrhosis has a worse prognosis than primary biliary cirrhosis and cirrhosis due to viral hepatitis [8]. Approximately 15% of patients with ascites die within the first 12 months after diagnosis and 44% within the first 60 months [9].

A recent survey reviewed 260 epidemiological studies on liver diseases in Europe. Alcohol consumption, viral hepatitis B and C, and metabolic syndromes related to obesity are the leading causes of cirrhosis and primary liver cancer here. Liver cirrhosis is responsible for around 170,000 deaths in Europe annually [10]. In the UK, liver disease is the 5th most common cause of death [11]. In the United States, chronic liver disease and cirrhosis ranked 12th among the leading causes of death in 2010 [12]. Figures are likely to be even higher in Asia and Africa, since childhood infection with HCV is more common there and the prevalence of cirrhosis in patients with chronic HCV increases with increasing duration of infection [13, 14].

In the course of the rising frequency of alcoholic and nonalcoholic fatty liver disease, a huge increase in the burden of liver diseases is expected over the next years [4]. In the UK, admissions to hospital and liver deaths are both rising at between 8 and 10% per year. Patients are presenting and dying from liver disease at an earlier age; the average age of death from liver disease is 59 years. Over the last 10 years there has been a 5-fold increase in the development of cirrhosis in 35- to 55-year-old patients [11]. Because of the increasing prevalence of chronic viral hepatitis and steatohepatitis and their high risk evolution toward end stage liver cirrhosis, preventive programs and early management of these conditions are considered as emerging health issues worldwide [14]. Treatments that may halt the progression of compensated to decompensated cirrhosis are currently being developed [15]. Liver transplantation, however, is the only option in patients with end stage disease. The cost of hepatic cirrhosis in terms of human suffering, hospital costs, and lost productivity is high [16, 17].

*Hepatic Cirrhosis in India*. Given the high incidence and prevalence of hepatitis B, hepatitis C, and fatty liver disease, hepatic cirrhosis is a common condition in India. Generally, Indian patients first seek help from conventional medicine, and in many cases liver transplantation is suggested to them. Actually, India would require up to 20,000 liver transplants per year. However, currently just 200–300 transplants per annum are possible within the framework of the Indian medical system. Liver transplantations are complex procedures; they require sophisticated infrastructures, expert medical teams, preservation of the transplant organs, expensive drugs, and prolonged stays in ICUs, all of which add significantly to the overall costs. A liver transplant requires about 50,000 USD, plus a lifelong commitment to immunosuppressants, costing about 2,500 USD per month, which is unmanageable for most Indian patients [18].

As to our observation, since the only curative yet unaffordable treatment option is liver transplantation in conventional medicine, in India, complementary and alternative medicine (CAM) treatments are often sought out instead, especially Ayurveda, the most established traditional whole medical system (WMS) in South Asia with a well developed infrastructure, recognized by the World Health Organization. (In the context of this paper, though useful for a reader not familiar with WMS Ayurveda, a detailed description of Ayurvedic medicine does not seem appropriate here. The interested reader may gather detailed information on the current infrastructure, institutional organization, education, practice, and research of Ayurveda from selected sources mentioned in Further Readings at the end of this paper.)

*CAM and Ayurveda Related Drug Research in Liver Diseases including Hepatic Cirrhosis*. Searching commonly accepted sources, only a paucity of scientifically evaluated CAM-options for the treatment of chronic liver diseases, especially of cirrhotic liver conditions, is retrievable.

S-Adenosylmethionine, polyenylphosphatidylcholine, betaine, and antioxidants like vitamins A, C, E, B6, and B12 as well as branched chain amino acids are examples of substances studied for their potential efficacy in cases of hepatic cirrhosis. Trials showed clinical improvements, but hardly any statistically significant improvements in liver function test [19–29].

For herbal drugs used in different fields of CAM limited data is available, which have shown beneficial effects on liver diseases in clinical trials [30–33]. Worldwide,* Silybum marianum* (L.) Gaertn. is currently the plant on which most research has been performed in the treatment of liver diseases [34–38]. From East Asia, the compound herbal drug TJ-9 is worth mentioning for chemoprevention in hepatocellular carcinoma, commonly prescribed as Xiao-Chai-Hu-Tang in China and as Sho-Saiko-To in Japan. It consists of an extract from the roots of* Scutellaria baicalensis* Georgi,* Glycyrrhiza glabra* L.,* Bupleurum falcatum* L., and* Panax ginseng* C. A. Mey [39].

CAM research on chronic liver diseases also incorporates drugs commonly used in Ayurveda. Nobel laureate Baruch Blumberg, awarded for his hepatitis B virus surface-antigen discovery, had already in the 1980s initiated research on* Phyllanthus niruri* Sensu Hook. F. non Linn. (*P. niruri*), seeking measures to prevent and treat hepatitis B [40]. Reviews in recent years come to conclusions varying from being positive to being indifferent regarding* P. niruri*'s effect in this condition [41–43]. As an example among animal studies, carbon tetrachloride- (CCl4-) induced increase of serum glutamic-pyruvic transaminase (ALT) and elevation of MDA in liver of mice are significantly lowered by* P. niruri in vivo* and the coincubation of isolated rat hepatocytes with* P. niruri in vitro* significantly inhibits CCl4-induced decrease of mobility of membrane of liver cells and increase of intracellular free Ca~(2+)([Ca~(2+)]_(i)) concentrations of liver cells. These results suggest that the antilipid peroxidation effect and protection of membranes through* P. niruri* may be related to its protective action against CCl4-induced liver injuries [44].

The positive action in chronic liver diseases of Liv 52, an Ayurvedic herbal compound preparation frequently used in India for chronic liver diseases, is well documented [45].

*Piper longum* Linn. fruit (*P. longum*), a traditional medicinal plant in Ayurveda, was examined for its hepatoprotective properties, concentrating on its main active ingredient piperine, which was reported to exert significant protection against acetaminophen-induced hepatotoxicity in mice [46].

Another medicinal plant in this context is* Picrorhiza kurroa* Royle ex Benth (*P. kurroa*). Cucurbitacin glycosides, isolated from the root of* P. kurroa*, exhibited liver protective and anti-inflammatory activities. Kutkin, a glycosidal bitter component of* P. kurroa* exhibited hepatoprotective activity in alcohol-induced hepatotoxicity in rats [47–51].

*Boerhavia diffusa* L. (*B. diffusa*) exhibits anti-inflammatory, hepatoprotective, and antioxidant effects and therefore can be considered as an option in cases of HCcA [52–55].

*Tephrosia purpurea* Pers. (*T. purpurea*) is widely used in the treatment of inflammation of the spleen and liver in the Ayurvedic tradition. Its powdered aerial parts prevent an elevation of GOT, GPT, and bilirubin levels [56, 57]. The cirrhotic and nodular changes induced by CCl4 were effectively prevented by* T. purpurea* showing that it might be acting by stabilizing cell membranes. These findings relate to an earlier study which reported that pretreatment of rats with Tefroli, a herbal product containing* T. purpurea* as one of the main ingredients, protected the rats against progress of hepatic fibrosis after chronic CCl4 intoxication [58].

Finally,* Eclipta alba* (L.) Hassk. (*E. alba*) holds a prominent position in the traditional use for hepatitis and spleen enlargements in Ayurveda and is considered to be a liver tonic [59]. The protective effect of* E. alba* on carbon tetrachloride-induced acute liver damage was also studied in 54 female guinea pigs in an experimental trial [60].

Hence, over the last years and decades, several research projects were dedicated to examining the effects of herbal drugs traditionally used in Ayurveda to support liver functions and treat common liver diseases. However, the effects of CAM herbal therapies on life-threatening chronic liver diseases have for the most part been evaluated on animal studies. Among the limited number of CAM clinical trials only a few deal with hepatic cirrhosis and so far no clinical study is known to the authors, which has directly evaluated Ayurvedic therapies in patients with HCcA.

*Rationale for the Ayurvedic Treatment Protocol of the Present Study*. On the basis of this preliminary evidence and as commonly practiced in the field of reverse pharmacology, treatment protocols incorporating empirical data from traditional treatment patterns of WMS Ayurveda can be considered as promising and ethically sound. In this sense, a standardized treatment protocol was developed from traditional Ayurvedic sources, supplemented by current research findings. It was implemented for several years at the P. D. Patel Ayurveda Hospital in Nadiad, India, before this observational study was conducted. Special attention was given to the proposition that medicinal plants exert more intense action, if embedded in complex treatment schemes. In Ayurveda, certain preparatory measures such as purificatory procedures (e.g., purgation) are considered to enhance the action of drugs. Also diet and lifestyle corrections are held to optimize the effect of any given treatment [61].

In current Ayurveda, HCcA is commonly related to the superordinate disease group of the so-called abdominal diseases (*udara roga*). Among eight types of “abdominal diseases” the most serious conditions are considered to be* jalodara* or* yakriddaludara*, which can be interpreted as different varieties of HCcA [62]. These nosological entities are described as having similar symptoms to another form of “abdominal disease” (*plihodara*) characterized by abdominal distention (due to splenomegaly), accompanied by weakness, anorexia, indigestion, constipation, excessive thirst, breathlessness, coughing, vomiting, cachexia, syncope or coma, and visible yellowish or indigo colored veins in the abdominal area [63].

The treatment protocol for the study was predominantly inspired by one of the most authoritative texts of Ayurveda (the* Caraka Samhita*). Initially, a procedure, which consists of a specific administration pattern of* P. longum* in increasing and tapering doses (*vardhamana-pippali-rasayana,* Table 2) [64], was performed on all patients. The microfine powder of dried* P. longum *fruit (family Piperaceae) was manufactured with the help of pulverizing machines by Sundar Ayurveda Pharmacy, that is, the pharmacy of the Pharmaceutical Department of J. S. Ayurved College, Nadiad, India, as all the following powders of dried plants. A large number of alkaloids and related compounds, the most abundant of which is piperine, together with methyl piperine, iperonaline, piperettine, asarinin, pellitorine, piperundecalidine, piperlongumine, and piperlonguminine are found in the fruit. According to classical Ayurveda, this procedure exerts a tissue-regenerating effect (*rasayana*), which can be interpreted as being hepatoprotective. The classical dosage patterns were found to be intolerable for patients of our hospital before the commencement of the study. Consequently, the study intervention was standardized to a lower dosage pattern.

The multidimensional approach of Ayurveda calls for certain “purification measures” in most diseases. Here, purgation induced by rhizomes and roots of* P. kurroa* was chosen in a dosage matched with the individual condition of each patient [65]. The powder of dried roots and rhizomes of* P. kurroa* (family Scrophulariaceae) mainly contains iridoid glycosides, cucurbitacins, unsaturated sterols/triterpenes, and polyphenols, especially kutkin, a bitter glycoside.

These “preparatory” treatments were embedded in a set of strict food and behavioral restrictions, based on classical Ayurvedic rationales [66]. Namely, a diet consisting only of boiled milk and maximum rest in an OPD setting were implemented.

Classically indicated drugs, like the powder of dried whole plants of* B. diffusa*,* T. purpurea*, and* E. alba*, were administered since the experimental studies cited above suggest a hepatoprotective action of these drugs and because (empirically) the combination of these drugs has been used for the treatment of HCcA over centuries in routine Ayurvedic therapy.* B. diffusa* (family Nyctaginaceae) contains mainly alanine, arachidic acid, aspartic acid, behenic acid, boeravinones A through F, boerhaavic acid, and borhavine. In* T. purpurea* (family Fabaceae) the presence of flavones, flavanones (e.g., purpurin) and prenylated flavonoids, chalcones, and rotenoids was found. From* E. alba*, Ecliptasaponin C, a new triterpenoid glucoside, was isolated together with daucosterol and stigmasterol-3-O-glucoside.

To promote diuresis, the herbal compound formulation* Punarnavadi-kvatha* [67] based on the whole plant of* B. diffusa* and the herbomineral combination* Shveta-Parpati* [68] were chosen. The ingredients of* Punarnavadi-kvatha* are listed in Table 3. The method of preparation follows the description of ancient Ayurvedic texts for decoction (*kvatha*). 10 g of the course powder of mentioned dried plants are boiled in 160 mL water in an open vessel on mild heat until it is reduced to one-fourth of the original water quantity (40 mL). This preparation is boiled fresh twice daily and administered orally after cooling down.* Shveta-Parpati* contains ashes of 1 g ammonium chloride [NH_4_Cl], 2 g potash alum [KAl (SO_4_)_2_12H_2_O], and 16 g potassium nitrate which are purified according to the classical method. All ingredients were triturated and afterwards deposited in a sealed earthen pot, which was kept in a furnace with fire for 8 hours. Then they were allowed to cool and powdered.

To this effect, the present nonrandomized, uncontrolled, single group, open-label observational clinical study scientifically explores effectiveness and safety of a treatment scheme, which generated clinically promising results in single cases of hepatic cirrhosis complicated by ascites in an Ayurvedic hospital.

## 2. Materials and Methods

### 2.1. Research Center

The P. D. Patel Ayurveda Hospital is a university teaching hospital of the J. S. Ayurved College, accredited by the Indian Government for under- and postgraduate Ayurveda education and run by a private charitable trust, the Mahagujarat Medical Society in Nadiad, India. All participants were screened and treated in both the in-patient department (IPD) and out-patient department (OPD) of the clinic.

The Institutional Ethics Committee of J. S. Ayurveda Mahavidyalaya, Nadiad, India, has given its permission to publish the data under the approval number 18/02-2014 (reference letter JSAM/14-15/26).

The laboratory consulted for all mentioned investigations is a part of the research center.

Adhering to standard operating procedures (SOP) and to classical descriptions all medicaments were purchased from Sundar Ayurveda Pharmacy, which is preparing all main Ayurvedic pharmaceuticals for the campus hospital. Collection of the used plant material complied with institutional, national, or international guidelines.

### 2.2. Patient Selection

The involved human subjects were all above 18 years of age. They were informed about the study details and gave consent to record and publish their data. Datasets were handled and displayed in a way which does not compromise anonymity or confidentiality or breach local data protection laws. Being an observational study, patients were chosen according to the inclusion and exclusion criteria.

Inclusion criteria were as follows: positive patient history and established diagnosis of HCcA according to international standards [15]; manifest clinical features, especially oedema, loss of appetite, general weakness, nausea and vomiting, increased measurement of abdominal girth (sign of ascites), and decreased urine output; manifest laboratory findings, especially hyperbilirubinemia, decreased serum albumin level, decreased albumin/globulin (A/G) ratio, raised serum alkaline phosphatase, raised liver transaminase enzymes, and confirmed abdominal ultrasound diagnosis of HCcA.

Exclusion criteria were as follows: hepatic cirrhosis due to cardiac causes, inherited metabolic causes, haemochromatosis and Wilson's disease; recent (≤3 month) life-threatening complications (like encephalopathy and excessive gastrointestinal bleeding), and other major comorbidities (like insulin-dependent diabetes mellitus, bleeding piles, manifest heart diseases, and renal failure). Female patients having pregnancy, postdelivery period, or lactation period and patients who were taking any psychiatric or other liver damaging medicines were also excluded.

Screening was performed between January 2007 and December 2010.

### 2.3. Study Protocol and Timeline

The study was planned to generate first exploratory data on the potential of Ayurvedic medicine for advanced liver diseases.

After screening, the main IPD observation period lasted for 6 weeks followed by an OPD follow-up of 18 weeks resulting in an overall 6-month observation period. During follow-up, patients were investigated for signs, symptoms, and laboratory findings (Table 1).

### 2.4. Therapy

The treatment conducted is standard care and best practice for the given disease at our hospital. All patients were treated according to the following treatment protocol standardized on the basis of classical Ayurvedic literature and traditional treatment patterns.

#### 2.4.1. Phase 1

At the beginning of the IPD period, finely powdered dried fruit of* P. longum* was administered orally in an increasing and tapering dose pattern twice daily with milk before meals. This classical procedure was performed for a period of 13 days (Table 2).

#### 2.4.2. Phase 2

On the morning of the 14th day, mild purgation was performed once, orally administering finely powdered dried rhizomes and roots of* P. kurroa* with warm water on an empty stomach in varying doses from 3 to 6 g according to the patients' individual sensitivity to purgatives (*koshtha*).

#### 2.4.3. Phase 3

For the next 4 weeks a number of Ayurvedic drugs were given orally twice daily after meals (Table 3).

#### 2.4.4. Diet and Lifestyle

In the IPD phase, a set of rigorous food (boiled pasteurized fat-free cow's milk) and behavioral restrictions (maximum rest in an IPD setting) was carried out. The participants were allowed to consume milk according to their digestive capacity varying from 2 to 3.5 litres per day. All other liquids and foods were prohibited.

#### 2.4.5. Conventional Medication

Patients on conventional diuretics (e.g., furosemide and spironolactone) were advised to continue their medication initially. Their dosage was gradually reduced according to decreasing oedema as well as abdominal girth and increase in urine output and eventually stopped as soon as the patient responded to the Ayurvedic treatment satisfactorily.

#### 2.4.6. Phase 4

During follow-up, patients were advised to continue all Ayurvedic medicaments at home except* P. longum*. In diet, they were instructed to take a special type of boiled beans (*Vigna radiata* (L.) Wilczek), rice, and boiled vegetables being easily digestible and nonslimy in consistency. Also nonsour fruits, like papaya, mango, sweet apple, and so forth, were permitted. All participants were strictly told to permanently abstain from all alcoholic beverages.

### 2.5. Assessment of Results

Treatment effects were measured by a predefined set of typical clinical features and laboratory parameters of HCcA. Additionally, the assessed outcomes included prognostic markers according to the Child-Pugh grade score.

#### 2.5.1. Clinical Features (Scores)

Following clinical features were selected as representative markers for the assessment of HCcA treatment during study visits (V0–V2): oedema, loss of appetite, general weakness, nausea and vomiting, abdominal girth, and daily urine output.

They were graded according to the score presented in Table 4. Changes in ascites were assessed by the measurement of abdominal girth and urine output in liters per day. All other investigations were also recorded before (V0) and after (V1) treatment and also during follow-up (V2).

Statistically, all outcomes were analyzed using a paired Student's test (“*t*” test). Explorative tests were not adjusted for multiple testing. A value of *p* < 0.05 was considered as statistically significant and *p* < 0.001 as statistically highly significant.

## 3. Results

### 3.1. Baseline (V0)

68 patients (48 males and 20 females) with HCcA were screened. Mean age was 46.45 years (<20 years of age: *n* = 2; 20–40 years of age: *n* = 25; >40 years of age: *n* = 41).

12 drop-outs happened due to personal reasons not related to the therapy, like social or family affairs (e.g., marriages or death of close family members, which are of major social relevance in South Asia). Overall, 56 patients' datasets were completely recorded.

Prolonged alcohol consumption and cryptogenic cirrhosis of liver were found as main causative factors in 26 patients. A positive patient history for different forms of hepatitis was present in 16 cases (Table 5). At the time of study entry (V0), all patients were treated with standard conventional medicine.

### 3.2. Treatment Effects

#### 3.2.1. Clinical Features

Oedema was reduced by 83.9%. Appetite increased by 64.7%. General weakness declined by 54.7%. Nausea and vomiting were relieved by 90.9%. Abdominal girth decreased by 19.7%. Urine output increased by 266.3%. All these clinical features were found to be statistically highly significant (*p* < 0.001) (Table 6).

#### 3.2.2. Laboratory

Accordingly, all relevant laboratory values improved in a statistically significant fashion; albumin (40.7%) and bilirubin (66.5%) (Table 7) were mostly pronounced.

#### 3.2.3. Child-Pugh Grade Score

**Figure 1:**
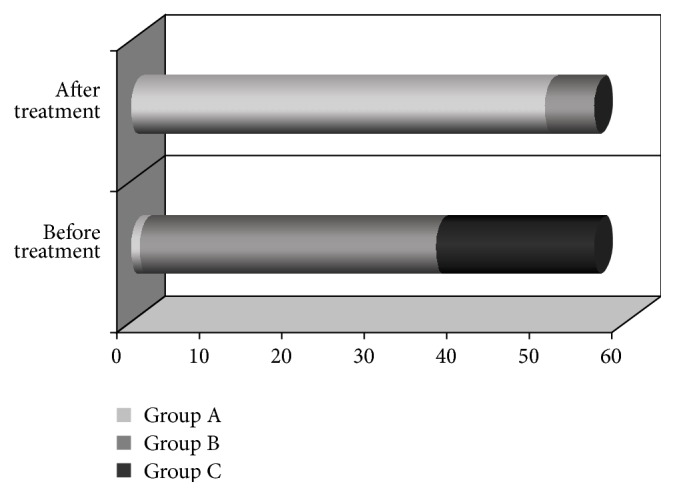
Effect on prognosis according to Child-Pugh grade score: overall change (*n* = 56).

At baseline (V0) 19 patients were graded in Group C (worst prognosis), 36 patients were graded in Group B (medium prognosis), and 1 was graded patient in Group A (best prognosis). After treatment (V1) 50 patients were found in Group A, 6 patients were found in Group B, and no patient was found in Group C (Figure 1).

56 patients completed the full 6-months treatment protocol. They adhered to the dietary and lifestyle advice and took their medicines regularly. During follow-up (V2) patients' general condition further improved and no setback of clinical features was observed. Liver function tests also further improved (see Table 7). With regard to the Child-Pugh grade score, 6 patients of Group B returned to Group A after completing the follow-up period; that is, all patients came to group A.

## 4. Discussion

In this study improvements in all clinical features and laboratory parameters were observed. The results were clinically relevant and statistically significant. Notably, albumin synthesis was initially impaired in all cases, but under this Ayurvedic treatment protocol albumin levels increased in all patients. Ascites could be reversed and controlled. None of the patients developed severe adverse effects or new complications and hence the treatment was found to be safe under the given conditions. Notably, in all participants conventional medication could be reduced or even withdrawn. Initially indicated invasive measures (e.g., liver transplantation) could be omitted, particularly through improvements of the Child-Pugh grade score in several patient cases. Patients were able to manage their routine everyday life affairs without becoming dependent on cost intensive conventional health care and maintenance measures within the observation period.

Hence, the treatment protocol can be considered as applicable and cost-effective and, probably, as a template for subsequent trials in this field. These results are promising and may be regarded as a novelty in clinical research on hepatic cirrhosis complicated by ascites, looking at an overall paucity of peer-reviewed publications on Ayurvedic treatment options in this field [61–66], and an absence of respective clinical study data available in internationally accessible medical databases.

From a conventional biomedical perspective, the probable mode of action of the study drugs may stem from their effects on specific metabolic liver functions and functions of associated organs, the details of which have still to be subject to future studies [40–60]. Taking the traditional Ayurvedic paradigms into account and Ayurveda's unique understanding of pathogenesis and treatment, more general terms should be used to explain their modes of action. Along the way, the challenges and problems arising in translational processes between systems' terminology remain an open field of study, in particular for qualitative research on WMS [67]. In summary, the study drugs' antioedemic, diuretic, immunomodulatory, rejuvenative, hepatosplenoprotective, and metabolism-promoting actions may be considered as being mainly responsible for the observed effects [68–75].

### 4.1. Limitations and Future Directions

The study is a nonrandomized, uncontrolled, single group, open-label observational clinical study. This design therefore bears major limitations. Various nonspecific effects, the setting, and the time course may have contributed to the beneficial course of the disease. Thus they may have influenced the results. Furthermore, changes in diet and the restricted alcohol consumption may have influenced the results. If the study therapies would have been studied with more patients and under controlled, randomized conditions, the effectiveness of the therapy could have been presented with more power, certainty, and accuracy. Another limitation is that patients with serious complications like severe gastrointestinal bleeding and hepatic encephalopathy were not included in this study. Though important for the Child-Pugh Score, patients with hepatic encephalopathy were a priori excluded during the screening process. Moreover, the inclusion of further assessment criteria like lipid profile data, PBMC transcripts profiles or miRNA evaluations, pre- and posttreatment USG, and liver biopsies would be helpful to give clues on histological changes in future studies. From a whole systems perspective it might also be considered a limitation that mind-body aspects of Ayurvedic medicine were not included in the treatment protocol [76].

Notably, the transferability of these results to Western clinical contexts, particularly looking at Ayurvedic treatment availability, economic aspects, and safety issues, remains another important field of discussion [77, 78]. For example, many of the therapies and drugs used in this study are not commonly available in Western countries, especially not in standardized and rigorously tested dosage forms, and, moreover, are usually much more expensive than in their countries of origin. Thus, the conclusions of this study remain to some extent regional to South Asia where Ayurveda is a recognized as a full-fledged and widely practiced system of medicine [79].

## 5. Conclusions

On the basis of these findings, the Ayurvedic treatment protocol could be a potentially safe and effective complement in the case of HCcA therapy. If replicated in larger trials, Ayurvedic medicine could represent a promising tool to postpone the need of liver transplantations, increase the QoL of patients with HCcA, and reduce overall treatment costs. However, randomized and controlled studies with larger numbers of patients are indicated to further evaluate the results of this Ayurvedic treatment protocol. Cross-cultural and interdisciplinary aspects should also be taken into account in future studies on WMS Ayurveda.

*Further Readings*

 WHO
   
http://www.who.int/dg/speeches/2008/20081107/en/
   
http://www.who.int/intellectualproperty/studies/B.Patwardhan2.pdf
   
http://www.who.int/medicines/areas/traditional/BenchmarksforTraininginAyurveda.pdf
   
http://whqlibdoc.who.int/hq/2000/WHO_EDM_TRM_2000.1.pdf
 Department of AYUSH, Ministry of Health and Family Welfare (AYUSH, CCIM)
   
http://www.indianmedicine.nic.in/
   
http://ccimindia.org/http://ccimindia.org/
   
http://www.nia.nic.in/
 Universities
   
http://www.ayurveduniversity.com/
   
http://www.bhu.ac.in/ims/ayurveda/ayur_highlights.htm
 Research
   
http://www.ccras.nic.in/
   
http://ayushportal.nic.in/
 Research Database
   
http://www.dharaonline.org/Forms/Home.aspx
 Journals
   
http://nopr.niscair.res.in/
   
http://www.ijaronline.com/
   
http://www.ancientscienceoflife.org/
   
http://www.ayujournal.org/
   
http://www.jaim.in/
   
http://bjournals.ub.rug.nl/index.php/ejim/
 Phytotherapy
   
http://www.nmpb.nic.in/
   
http://nopr.niscair.res.in/
   
http://www.medicinalplants.in/

## Figures and Tables

**Table 1:** Timeline of the study.

Phase	Event	Measure	Week
Prephase	Screening	(i) Inclusion and exclusion criteria	Week 0
Main observation period (IPD)	Baseline visit (V0)	(i) Clinical and laboratory investigations (ii) Admission to IPD	Week 1 (day 1)
Phase 1		(i) *Piper longum* Linn. administration (specific dose pattern) (ii) Strict diet and lifestyle measures	Weeks 1 & 2 (days 1–13)
Phase 2		(i) Purgation (ii) Strict diet and lifestyle measures	Week 2 (day 14)
Phase 3		(i) Drug administration (ii) Strict diet and lifestyle measures	Weeks 3–6
	First visit (V1)	(i) Clinical and laboratory investigations (ii) Discharge from IPD	Week 7
Follow-up (OPD) Phase 4		(i) Continued drug administration (ii) Diet and lifestyle measures	Weeks 7–24
	Second visit (V2)	(i) Clinical and laboratory investigations	Week 24

**Table 2:** Dose pattern of *Piper longum* Linn.

Day	1	2	3	4	5	6	7	8	9	10	11	12	13
Dose [g] twice daily					5	5	5	5	5				
				4						4			
			3								3		
		2										2	
	1												1

**Table 3:** Treatment protocol for Ayurvedic drugs.

Number	Botanical name	Ayurvedic name	Content ratio	Part used	Form	Dose
(1)	Compound decoction	*Punarnavadi-kvatha *		Various	Coarse powder/decoction	10 g/40 mL
(a)	*Boerhavia diffusa* L. L. Linn.	*Punarnava *	1 part	Whole plant	Coarse powder	
(b)	*Berberis aristata* DC.	*Daruharidra *	1 part	Whole plant	Coarse powder	
(c)	*Cyperus rotundus* L.	*Musta *	1 part	Root	Coarse powder	
(d)	*Curcuma longa* L.	*Haridra *	1 part	Rhizome	Coarse powder	
(e)	*Azadirachta indica* A. Juss.	*Nimba *	1 part	Bark	Coarse powder	
(f)	*Tinospora cordifolia* (Willd.) Hook. F. & Thoms.	*Guduci *	1 part	Stem	Coarse powder	
(g)	*Zingiber officinale* Rosc.	*Shunthi *	1 part	Rhizome	Coarse powder	
(h)	*Picrorhiza kurroa* Royle ex Benth.	*Katuki *	1 part	Root	Coarse powder	

(2)	Compound powder				Fine powder	5.5 g
(a)	*Tephrosia purpurea* Pers. Linn. Pers.	*Sharapunkha *		Root	Fine powder	2 g
(b)	*Phyllanthus niruri* Sensu Hook. F. non Linn.	*Bhumyamalaki *		Whole plant	Fine powder	3 g
(c)	Herbomineral combination	*Shveta-Parpati *			Ultrafine powder (ash)	0.5 g
(I)	Ammonium chloride [NH_4_Cl]	*Navasara *	1 part		Ultrafine powder (ash)	
(II)	Potassium nitrate [KNO_3_]	*Suryakshara *	16 parts		Ultrafine powder (ash)	
(III)	Potash alum [KAl(SO_4_)_2_12H_2_O]	*Sphatika *	2 parts		Ultrafine powder (ash)	

(3)	*Eclipta alba* (L.) Hassk.	*Bhrngaraja *		Whole plant	Fine powder	3 g

**Table 4:** Assessed clinical features' score (Grade 0–3).

Number	Symptom	Grade 0	Grade 1	Grade 2	Grade 3
(1)	Oedema	No oedema	Slight oedema on lower extremities	Severe oedema on lower extremities	Anasarca
(2)	Loss of appetite	Good appetite	Mild loss of appetite	Moderate loss of appetite	Complete loss of appetite
(3)	General weakness	No weakness	Mild weakness	Moderate weakness	Severe weakness
(4)	Nausea and vomiting	Absent	Occasional	Once or twice a week	Daily
(5)	Abdominal girth [cm]	Grading per [cm]-scale			
(6)	Urine output [L/day]	Grading per [L]-scale			

**Table 5:** Baseline (V0) characteristics: underlying causes of HCcA (*n* = 68).

Basic cause (diagnosis)	Number of patients	Percentage [%]
Prolonged alcohol consumption	26	38
Alcohol and Hepatitis B	6	9
Hepatitis B	8	12
Hepatitis C	2	3
Cryptogenic cirrhosis	26	38

**Table 6:** Changes in clinical symptoms from baseline (V0) to week 7 (V1).

Number	Symptom score	Mean score		Improvement [%]	*p* value
		Baseline (V0)	Week 7 (V1)		
(1)	Oedema score	2.01 ± 0.68	0.33 ± 0.57	83.87 ± 0.62	<0.001
(2)	Loss of appetite score	2.62 ± 0.53	0.92 ± 0.52	64.70 ± 0.51	<0.001
(3)	General weakness score	2.67 ± 0.47	1.21 ± 0.5	54.67 ± 0.58	<0.001
(4)	Nausea and vomiting score	1.29 ± 0.59	0.12 ± 0.33	90.90 ± 0.4	<0.001
(5)	Abdominal girth [cm]	89.9 ± 10.6	72.1 ± 9.76	19.7 ± 7.17	<0.001
(6)	Urine output [liter/per day]	0.45 ± 0.29	1.63 ± 0.58	266.32 ± 0.5	<0.001

**Table 7:** Changes in clinical laboratory: baseline (V0), week 7 (V1), and follow-up (V2).

Investigation	Mean score	Improvement in percent [%]				*p* value
	Baseline (V0)	Week 7 (V1)	After 6 months (V2)	After Week 7 (V1)	Between V1 and V2	
Serum bilirubin [mg/dL]	3.74 ± 4.59	1.25 ± 1.31	0.94 ± 0.63	66.5 ± 4.04	25.63 ± 0.70	<0.001
Serum albumin [g/dL]	2.71 ± 0.67	3.32 ± 0.45	3.43 ± 0.28	22.43 ± 0.54	3.27 ± 0.29	<0.001
Serum albumin/serum globulin [A/G]	0.73 ± 0.20	1.03 ± 0.20	1.17 ± 0.17	40.65 ± 0.19	13.33 ± 0.17	<0.001
Serum alkaline phosphatase [IU/L]	318.3 ± 102.2	210.8 ± 53.05	192.15 ± 29.42	33.8 ± 76.37	8.37 ± 30.60	<0.001
Serum globulin [g/dL]	3.77 ± 0.62	3.27 ± 0.43	2.96 ± 0.32	13.3 ± 0.51	9.33 ± 0.37	<0.001
Hemoglobin [g/dL]	8.78 ± 1.90	10.36 ± 1.52	11.33 ± 1.47	17.98 ± 0.93	9.36 ± 1.06	<0.001
Serum GPT [IU/L]	58.41 ± 44.38	40.62 ± 27.6	30.62 ± 9.07	30.5 ± 27.39	24.60 ± 21.62	<0.001
Serum GOT [IU/L]	84.52 ± 50.52	52.6 ± 31.21	44.27 ± 21.81	37.8 ± 34.92	14.67 ± 14.63	<0.001

## Abbreviations

A/G: Albumin/globulin ratio
ALT: Serum glutamic-Pyruvic Transaminase
*B. diffusa*: * Boerhavia diffusa* L.
CAM: Complementary and alternative medicine
CCI4: Carbon tetrachloride
*E. alba*: * Eclipta alba* (L.) Hassk.
HCcA: Hepatic cirrhosis complicated by ascites
HCV: Hepatitis C virus
ICU: Intensive care unit
IPD: In-patient department
OPD: Out-patient department
*P. longum*: * Piper longum*
*P. kurroa*: * Picrorhiza kurroa*
*P. niruri*: * Phyllanthus niruri* Sensu Hook. F. non Linn.
QoL: Quality of life
SOP: Standard operating procedures
T. purpurea: Tephrosia purpurea Pers.
V0: Baseline visit
V1: First visit (week 7)
V2: Second visit (follow up, after 6 months)
WHO: World Health Organization
WMS: Whole medical system.

## References

[1] Garcia-Tsao G., Lim J. K. (2009). Management and treatment of patients with cirrhosis and portal hypertension: recommendations from the department of veterans affairs, Hepatitis C Resource Center Program and the National Hepatitis C Program. *The American Journal of Gastroenterology*.

[2] D'Amico G., Garcia-Tsao G., Pagliaro L. (2006). Natural history and prognostic indicators of survival in cirrhosis: a systematic review of 118 studies. *Journal of Hepatology*.

[3] Ginés P., Quintero E., Arroyo V. (1987). Compensated cirrhosis: natural history and prognostic factors. *Hepatology*.

[4] Fisher N. C., Hanson J., Phillips A., Rao J. N., Swarbrick E. T. (2002). Mortality from liver disease in the West Midlands, 1993–2000: observational study. *British Medical Journal*.

[5] D'amico G., Morabito A., Pagliaro L., Marubini E. (1986). Survival and prognostic indicators in compensated and decompensated cirrhosis. *Digestive Diseases & Sciences*.

[6] Llach J., Gines P., Arroyo V. (1988). Prognostic value of arterial pressure, endogenous vasoactive systems, and renal function in cirrhotic patients admitted to the hospital for the treatment of ascites. *Gastroenterology*.

[7] Lucena I. M., Andrade R. J., Tognoni G., Hidalgo R., de la Cuesta F. (2002). Multicenter hospital study on prescribing patterns for prophylaxis and treatment of complications of cirrhosis. *European Journal of Clinical Pharmacology*.

[8] Sørensen H. T., Thulstrup A. M., Mellemkjar L. (2003). Long-term survival and cause-specific mortality in patients with cirrhosis of the liver: a nationwide cohort study in Denmark. *Journal of Clinical Epidemiology*.

[9] Planas R., Montoliu S., Ballesté B. (2006). Natural history of patients hospitalized for management of cirrhotic ascites. *Clinical Gastroenterology and Hepatology*.

[10] Blachier M., Leleu H., Peck-Radosavljevic M., Valla D.-C., Roudot-Thoraval F. (2013). The burden of liver disease in Europe: a review of available epidemiological data. *Journal of Hepatology*.

[11] http://www.bsg.org.uk/clinical/commissioning-report/management-of-patients-with-chronic-liver-diseases.html.

[12] Murphy S. L., Xu J., Kochanek K. D. (2013). Deaths: final data for 2010. *National Vital Statistics Reports*.

[13] D'Souza R., Glynn M. J., Ushiro-Lumb I. (2005). Prevalence of hepatitis C-related cirrhosis in elderly asian patients infected in childhood. *Clinical Gastroenterology and Hepatology*.

[14] Ullah F., Khan S., Afridi A. K., Rahman S. U. (2012). Frequency of different causes of cirrhosis liver in local population. *Gomal Journal of Medical Sciences*.

[15] Grattagliano I., Ubaldi E., Bonfrate L., Portincasa P. (2011). Management of liver cirrhosis between primary care and specialists. *World Journal of Gastroenterology*.

[16] Schuppan D., Afdhal N. H. (2008). Liver cirrhosis. *The Lancet*.

[17] Minino A. M., Heron M. P., Murphy S. L., Kochanek K. D. (2007). Deaths: final data for 2004. *National Vital Statistics Reports*.

[18] Suryanarayan D. India needs 20,000 cadaver livers a year: Mohamed Rela. http://hepatitiscresearchandnewsupdates.blogspot.de/2010/06/india-needs-20000-cadaver-livers-year.html.

[19] Abittan C. S., Lieber C. S. (1999). Alcoholic liver disease. *Current Treatment Options in Gastroenterology*.

[20] Mato J. M., Cámara J., de Paz J. F. (1999). S-adenosylmethionine in alcoholic liver cirrhosis: a randomized, placebo-controlled, double-blind, multicenter clinical trial. *Journal of Hepatology*.

[21] Chitturi S., Farrell G. C. (2000). Herbal hepatotoxicity: an expanding but poorly defined problem. *Journal of Gastroenterology and Hepatology*.

[22] Angulo P., Lindor K. D. (2001). Treatment of nonalcoholic fatty liver: present and emerging therapies. *Seminars in Liver Disease*.

[23] Liu C.-T., Chuang P.-T., Wu C.-Y., Weng Y.-M., Chen W., Tseng C.-Y. (2006). Antioxidative and *in vitro* hepatoprotective activity of *Bupleurum kaoi* leaf infusion. *Phytotherapy Research*.

[24] Rambaldi A., Gluud C. (2006). S-adenosyl-L-methionine for alcoholic liver diseases. *The Cochrane Database of Systematic Reviews*.

[25] Cave M., Deaciuc I., Mendez C., Song Z., Joshi-Barve S., McClain C. (2007). Nonalcoholic fatty liver disease: predisposing factors and the role of nutrition. *The Journal of Nutritional Biochemistry*.

[26] Lirussi F., Azzalini L., Orando S., Orlando R., Angelico F. (2007). Antioxidant supplements for non-alcoholic fatty liver disease and/or steatohepatitis. *Cochrane Database of Systematic Reviews*.

[27] Moriarty K. J., Platt H., Crompton S. (2007). Collaborative care for alcohol-related liver disease. *Clinical Medicine*.

[28] Nakaya Y., Okita K., Suzuki K. (2007). BCAA-enriched snack improves nutritional state of cirrhosis. *Nutrition*.

[29] Urata Y., Okita K., Korenaga K., Uchida K., Yamasaki T., Sakaida I. (2007). The effect of supplementation with branched-chain amino acids in patients with liver cirrhosis. *Hepatology Research*.

[30] Luper S. (1998). A review of plants used in the treatment of liver disease: part 1. *Alternative Medicine Review*.

[31] Langmead L., Rampton D. S. (2001). Review article: herbal treatment in gastrointestinal and liver disease—benefits and dangers. *Alimentary Pharmacology & Therapeutics*.

[32] Sharma A., Sharma M. S., Mishra A., Sharma S., Kumar B., Bhandari A. (2011). Review on Thar plants used in liver diseases. *International Journal on Research Methodologies in Physics and Chemistry*.

[33] Del Prete A., Scalera A., Iadevaia M. D. (2012). Herbal products: benefits, limits, and applications in chronic liver disease. *Evidence-Based Complementary and Alternative Medicine*.

[34] Salmi H. A., Sarna S. (1982). Effect of silymarin on chemical, functional, and morphological alterations of the liver. A double-blind controlled study. *Scandinavian Journal of Gastroenterology*.

[35] Hruby K., Csomos G., Fuhrmann M., Thaler H. (1983). Chemotherapy of *Amanita phalloides* poisoning with intravenous silibinin. *Human Toxicology*.

[36] Trinchet J. C., Coste T., Lévy V. G. (1989). Treatment of alcoholic hepatitis with silymarin. A double-blind comparative study in 116 patients. *Gastroentérologie Clinique et Biologique*.

[37] Ferenci P., Dragosics B., Dittrich H. (1989). Randomized controlled trial of silymarin treatment in patients with cirrhosis of the liver. *Journal of Hepatology*.

[38] Parés A., Planas R., Torres M. (1998). Effects of silymarin in alcoholic patients with cirrhosis of the liver: results of a controlled, double-blind, randomized and multicenter trial. *Journal of Hepatology*.

[39] Oka H., Yamamoto S., Kuroki T. (1995). Prospective study of chemoprevention of hepatocellular carcinoma with sho-saiko-to (TJ-9). *Cancer*.

[40] Venkateswaran P. S., Millman I., Blumberg B. S. (1987). Effects of an extract from *Phyllanthus niruri* on hepatitis B and woodchuck hepatitis viruses: in vitro and in vivo studies. *Proceedings of the National Academy of Sciences of the United States of America*.

[41] Liu J., Lin H., McIntosh H. (2001). Genus Phyllanthus for chronic hepatitis B virus infection: a systematic review. *Journal of Viral Hepatitis*.

[42] Xia Y., Luo H., Liu J. P., Gluud C. (2011). Phyllanthus species for chronic hepatitis B virus infection. *Cochrane Database of Systematic Reviews*.

[43] Xia Y., Luo H., Liu J. P., Gluud C. (2013). Phyllanthus species versus antiviral drugs for chronic hepatitis B virus infection. *Cochrane Database of Systematic Reviews*.

[44] Zhou S., Xu C., Zhou N. (1997). Mechanism of protective action of *Phyllanthus urinaria* L. against injuries of liver cells. *China Journal of Chinese Materia Medica*.

[45] Fleig W. W., Morgan M. Y., Hölzer M. A. (1997). The ayurvedic drug LIV 52 in patients with alcoholic cirrhosis. Results of a prospective, randomized, double-blind, placebo-controlled clinical trial. *Journal of Hepatology*.

[46] Sabina E. P., Souriyan A. D. H., Jackline D., Rasool M. K. (2010). Piperine, an active ingredient of black pepper attenuates acetaminophen-induced hepatotoxicity in mice. *Asian Pacific Journal of Tropical Medicine*.

[47] Pandey B. L., Das P. K. (1989). Immunopharmacological studies on Picrorhiza kurroa royle-ex-benth part IV: cellular mechanisms of anti-inflammatory action. *Indian Journal of Physiology and Pharmacology*.

[48] Floersheim G. L., Bieri A., Koenig R., Pletscher A. (1990). Protection against *Amanita phalloides* by the iridoid glycoside mixture of *Picrorhiza kurroa* (kutkin). *Agents and Actions*.

[49] Shukla B., Visen P. K. S., Patnaik G. K., Dhawan B. N. (1991). Choleretic effect of picroliv, the hepatoprotective principle of *Picrorhiza kurroa*. *Planta Medica*.

[50] Rastogi R., Saksena S., Garg N. K., Kapoor N. K., Agarwal D. P., Dhawan B. N. (1996). Picroliv protects against alcohol-induced chronic hepatotoxicity in rats. *Planta Medica*.

[51] Vaidya A. B., Antarakar D. S., Doshi J. C. (1996). *Picrorhiza kurroa* Royle-ex-Benth as a hepatoprotective agent—experimental and clinical studies. *Journal of Postgraduate Medicine*.

[52] Singh R. H., Udupa K. N. (1972). Studies on the Indian indigenous drug Punarnava (*Boerhaavia diffusa* L.) Part IV. Preliminary controlled clinical trial in nephrotic syndrome. *Journal of Research in Indian Medicine*.

[53] Manu K. A., Kuttan G. (2009). Punarnavine induces apoptosis in B16F-10 melanoma cells by inhibiting NF-*κ*B signaling. *Asian Pacific Journal of Cancer Prevention*.

[54] Manu K. A., Kuttan G. (2009). Immunomodulatory activities of Punarnavine, an alkaloid from *Boerhaavia diffusa*. *Immunopharmacology and Immunotoxicology*.

[55] Olaleye M. T., Akinmoladun A. C., Ogunboye A. A., Akindahunsi A. A. (2010). Antioxidant activity and hepatoprotective property of leaf extracts of *Boerhaavia diffusa* Linn against acetaminophen-induced liver damage in rats. *Food and Chemical Toxicology*.

[56] Ramamurthy M., Srinivasan M. (1993). Hepatoprotective effect of *Tephrosia purpurea* in experimental animals. *Indian Journal of Pharmacology*.

[57] Khatri A., Garg A., Agrawal S. S. (2009). Evaluation of hepatoprotective activity of aerial parts of *Tephrosia purpurea* L. and stem bark of *Tecomella undulata*. *Journal of Ethnopharmacology*.

[58] Chauhan C. K., Nanivadekar S. A., Billimoria F. R. (1992). Effect of a herbal hepatoprotective product on drug metabolism in patients of cirrhosis and hepatic enzyme function in experimental liver damage. *Indian Journal of Pharmacology*.

[59] Prabu K., Kanchana N., Sadiq A. M. (2011). Hepatoprotective effect of *Eclipta alba* on paracetamol induced liver toxicity in rats. *Journal of Microbiology and Biotechnology Research*.

[60] Ma-Ma K., Nyunt N., Tin K. M. (1978). The protective effect of Eclipta alba on carbon tetrachloride-induced acute liver damage. *Toxicology and Applied Pharmacology*.

[61] Rohilla R., Garg T., Goyal A. K., Rath G. (2014). Herbal and polymeric approaches for liver-targeting drug delivery: novel strategies and their significance. *Drug Delivery*.

[62] Palbag S., Dey B. K., Singh N. K. (2014). Ethnopharmacology, phytochemistry and pharmacology of *Tephrosia purpurea*. *Chinese Journal of Natural Medicines*.

[63] Manvar D., Mishra M., Kumar S., Pandey V. N. (2012). Identification and evaluation of anti Hepatitis C virus phytochemicals from *Eclipta alba*. *Journal of Ethnopharmacology*.

[64] Donepudi A. C., Aleksunes L. M., Driscoll M. V., Seeram N. P., Slitt A. L. (2012). The traditional ayurvedic medicine, *Eugenia jambolana* (Jamun fruit), decreases liver inflammation, injury and fibrosis during cholestasis. *Liver International*.

[65] Dhiman R. K., Chawla Y. K. (2005). Herbal medicines for liver diseases. *Digestive Diseases and Sciences*.

[66] Patel J. C. (2001). Cirrhosis of liver with ascites treatment based on principles of ayurved. *Indian Journal of Medical Sciences*.

[67] Witt C. M., Michalsen A., Roll S. (2013). Comparative effectiveness of a complex Ayurvedic treatment and conventional standard care in osteoarthritis of the knee—study protocol for a randomized controlled trial. *Trials*.

[68] Sharma R. K., Dash B. (2000). *Agnivesha's Caraka Samhita*.

[69] Sharma R. K., Dash B. (2000). *Agnivesha's Caraka Samhita*.

[70] Sharma R. K., Dash B.

[71] Sharma R. K., Dash B.

[72] Sharma R. K., Dash B.

[73] Sharma R. K., Dash B.

[74] Sen G.

[75] Achary Y. T.

[76] Kessler C., Wischnewsky M., Michalsen A., Eisenmann C., Melzer J. (2013). Ayurveda: between religion, spirituality, and medicine. *Evidence-Based Complementary and Alternative Medicine*.

[77] Saper R. B., Kales S. N., Paquin J. (2004). Heavy metal content of ayurvedic herbal medicine products. *Journal of the American Medical Association*.

[78] Saper R. B., Phillips R. S., Sehgal A. (2008). Lead, mercury, and arsenic in US- and Indian-manufactured Ayurvedic medicines sold via the internet. *The Journal of the American Medical Association*.

[79] WHO http://apps.who.int/medicinedocs/documents/s17552en/s17552en.pdf.

